# Genetically engineered pigs for xenotransplantation: Hopes and challenges

**DOI:** 10.3389/fcell.2022.1093534

**Published:** 2023-01-12

**Authors:** Jiahui Xi, Wei Zheng, Min Chen, Qingjian Zou, Chengcheng Tang, Xiaoqing Zhou

**Affiliations:** Guangdong Provincial Key Laboratory of Large Animal Models for Biomedicine, South China Institute of Large Animal Models for Biomedicine, School of Biotechnology and Health Science, Wuyi University, Jiangmen, China

**Keywords:** genetically engineered pigs, xenotransplantation, immunologic barriers, interspecies chimeras, porcine endogenous retroviruses (PERV)

## Abstract

The shortage of donor resources has greatly limited the application of clinical xenotransplantation. As such, genetically engineered pigs are expected to be an ideal organ source for xenotransplantation. Most current studies mainly focus on genetically modifying organs or tissues from donor pigs to reduce or prevent attack by the human immune system. Another potential organ source is interspecies chimeras. In this paper, we reviewed the progress of the genetically engineered pigs from the view of immunologic barriers and strategies, and discussed the possibility and challenges of the interspecies chimeras.

## 1 Introduction

**FIGURE 1:**
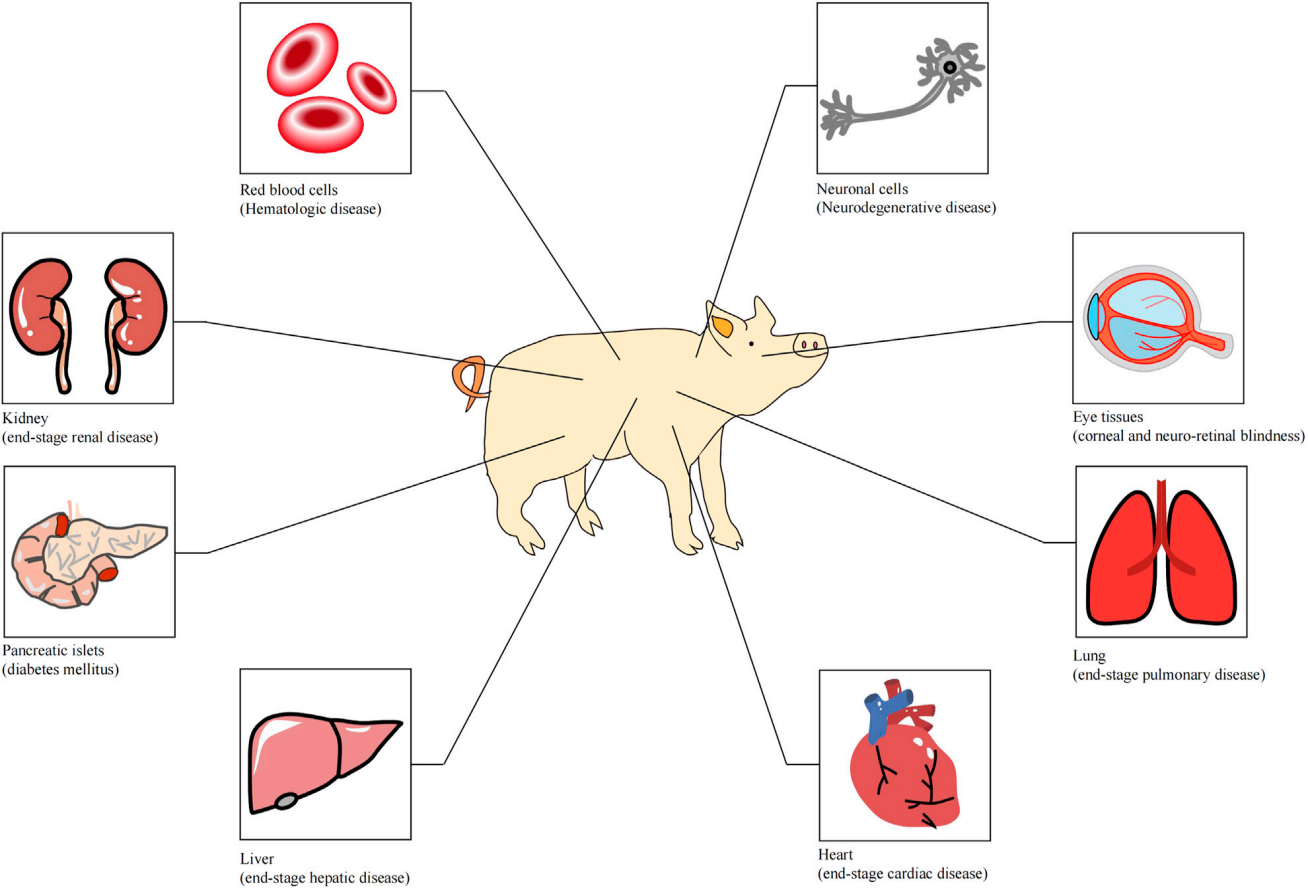
Tissues or organs that can be used for xenotransplantation (Ekser et al. 2012).

Organ transplantation is one of the greatest medical achievements in the 20th century and has saved the lives of thousands of patients who were suffering from organ failure. With the rapid developments in surgical techniques and the utilization of immunosuppressive agents, allotransplantation has become the only available treatment for end-stage organ failure in biomedicine. However, the disparity between organ supply and the demand for human organs is a bottleneck for clinical allotransplantation. According to the statistical results of United States Government Information on Organ Donation and Transplantation, 105,000 patients are currently waiting for transplants in the United States at present, and approximately 17 people die daily whilst waiting for a transplant. The organ supply in developed countries can only meet 15% of the needs, and about one-fifth of patients die while waiting. In China, the ratio of patients on the waiting list to the transplant recipients is 30:1.6 (Pan et al., 2019). Research on xenotransplantation was brought into being because of the increasingly arresting contradiction between organ supply and the demand for organ resources. Many disorders could be treated with clinical xenotransplantation (Figure 1) (Ekser et al., 2012). At present, various large animals have been used in xenotransplantation, including pigs, monkeys, chimpanzees and baboons. Pigs are considered the most ideal organ xenograft donor because their organ size, physiological metabolism and immune system are similar to those of human beings. However, natural immunologic barriers exist between pig organs and human organs. So how can suitable pig organs be produced for xenotransplantation?

One approach is to genetically modify the donor pigs to reduce or prevent attack by the human immune system, and thus achieve the compatibility with the human body. Another possible approach is the use of interspecies chimeras, in which human stem cells are made chimeric with pigs with specific organ defects, and then developed into a particular organ under a human micro-environment.

## 2 Genetically modified pigs provide organs for xenotransplantation

In recent years, some non-cellular tissues from pigs have been used in clinical applications, such as pig cardiac valves for plastic surgery, small intestinal submucosa for bladder repair, and dermis for extensive burns (Laurencin and El-Amin, 2008). Meanwhile, the use of pigs with one or more immune-related genes modified has made a significant advance in solid organ transplantation from pigs to other large animals, and has resulted in remarkably long survival times for both recipients and grafts (Cooper, 2003). The survival time of neurons from *CTLA4-Ig* modified pigs reached 549 days after xenotransplantation (Badin et al., 2013). Islet cells xenografted from *hCD46* transgenic pigs survived for 950 days (Shin et al., 2016). The use of genetically modified pigs has greatly addressed the immune barriers to xenotransplantation. Hyperacute rejection and acute cellular rejection have been nearly overcome (Kuwaki et al., 2005), and results from these studies have greatly contributed to research on acute vascular rejection and chronic rejection. With the discovery of an increasing number of immune-related key genes and the rapid development of efficient gene modification methods, the clinical application of xenotransplantation using genetically modified pigs as donors in preclinical application is becoming more feasible (Table 1). The immune barriers to xenotransplantation can be overcome *via* several genetic modification strategies that address the different modes of rejection discussed below.

**TABLE 1:** Genetically modified pigs for xenotransplantation.

	Target genes	Gene modifications	Functions	References
Incompatible epitopes	*GGTA1*	Gene knockout	Deletion of α-Gal epitope	Cheng et al. (2016); Dai et al. (2002); Feng et al. (2016); Gao et al. (2017); Kolber-Simonds et al., 2004; Lai et al. (2002); Phelps et al., 2003)
	*GGTA1/hCD46*	Gene knockout & Transgenic	Deletion of α-Gal epitope, inactivation of complement system	Dong et al. (2017); Mohiuddin et al. (2010)
	*GGTA1/β4GalNT2*	Gene knockout	Deletion of α-Gal epitope and SDa blood group	Adams et al. (2018)
	*GGTA1/CMAH*	Gene knockout	Deletion of α-Gal epitope and N-glycolylneuraminic acid	Lai et al. (2002); Li et al. (2015); Lutz et al. (2013)
	*GGTA1/β4GalNT2/CMAH*	Gene knockout	Deletion of α-Gal epitope, N-glycolylneuraminic acid and SDa blood group	Estrada et al. (2015); Zhang et al. (2018)
Immunological cell	*hCTLA4-Ig*	Transgenic	Reduction of the proliferative response of human T lymphocytes	(badin et al., 2010; Martin et al. (2005)
	*GGTA1/HLA-G*	Gene knockout & Transgenic	Reduction of NK cell attack	Zhou et al. (2022)
	*CIITA-DN*	Transgenic	Suppression of T-cell activation	Hara et al. (2013)
	*HLA-E/β2m*	Transgenic	Protection of against human NK cell-mediated cytotoxicity	Weiss et al. (2009)
	*A20*	Transgenic	Reduction of apoptosis and inflammatory stimuli	Oropeza et al. (2009)
Complement system	*hCD46*	Transgenic	Inactivation of complement system	(Diamond et al., 2001; van der Windt et al. (2009)
	*hCD55*	Transgenic	Acceleration of complement decay	(Baldan et al., 2004)
PERV	PERV	Gene knockout	Inactivation of porcine endogenous retrovirus	Niu et al. (2017)
Multiple system	PERV/*GGTA1/CMAH/B4GALNT2/hCD46/hCD55/hCD59/hTHBD/hTFPI/hCD39/hB2M/HLA-E/hCD47*	Gene knockout & Transgenic	Inactivation of porcine endogenous retrovirus, deletion of α-Gal epitope, inactivation of complement system	Yue et al. (2021)

### 2.1 Hyperacute rejection

This type of rejection is mediated by the xenoreactive natural antibodies (XNAs) from the recipient and occur within minutes or hours after the restoration of xenograft blood circulation. XNAs bind to the xenoantigens of the xenograft and activate the classical complement pathway in the recipient, resulting in interstitial hemorrhage, edema and thrombosis of the xenograft, and finally leading to inactivation and necrosis of the xenograft within a few minutes or hours (Shimizu and Yamada, 2006). After more than a century of research, various methods to prevent hyperacute rejection have been developed. The first and most essential method is to reduce or eliminate the expression of galactose-α1,3-galactose (α-Gal), which is the main xenoantigen recognized by XNAs. α-Gal is expressed by α-1, 3-galactosyltransferase (*GGTA1*) gene and functions in pigs, but not in human beings, apes, or Old World monkeys (Good et al., 1992; Cooper et al., 1993). In 2002, Lai et al. and Dai et al. first produced clone pigs with *GGTA1* gene deficient, which was seen as a milestone of xenotransplantation field (Dai et al., 2002; Lai et al., 2002). Subsequently, an increasing number of GTKO (*GGTA1* knock out) pigs have been generated and used in xenotransplantation research (Cheng et al., 2016; Feng et al., 2016). When xenograft organs from GTKO pigs were transplanted into non-human primates, the survival times of the xenograft and the recipient were significantly prolonged, and the frequency of hyperacute rejection was significantly reduced or even disappeared. In other studies, a heart from a GTKO/*hTBM/hCD46* pig survived for 945 days after transplantation into baboons (Mohiuddin et al., 2016), and kidney xenografts from a GTKO/*hCD55* pig survived for 499 days after transplantation into rhesus macaque (Kim et al., 2019).

Except for α-Gal, two other non-Gal epitopes present an additional barrier to xenotransplantation. One is N-glycolylneuraminic acid (Neu5Gc), which is encoded by CMP-N-acetylneuraminic acid hydroxylase (*CMAH*) gene (Song et al., 2010). The other is the SDa blood group, which is produced by beta-1,4-N-acetyl-galactosaminyltransferase 2 (β4GALNT2) (Byrne, Ahmad-Villiers, Du, & McGregor, 2018). Attempts have been made to overcome these new xenoantigens by developing new transgenic pigs. The double or triple knockout pigs, which eliminate a-Gal, Neu5Gc, and the SDa epitopes, exhibited reduced human antibody binding *in vitro* (Lutz et al., 2013; Estrada et al., 2015; Li et al., 2015; Adams et al., 2018).

Another method for preventing hyperacute rejection is to inhibit complement response. Hyperacute rejection is mainly caused by the activation of complement pathway when XNAs bind to α-Gal. Thus, inhibiting the complement response can achieve the same effect. Numerous studies have shown that the frequency of hyperacute rejection during xenotransplantation was significantly reduced when transgenic pigs with human complement regulatory proteins (hCRPs) such as CD46, CD55, or CD59 were used (Huang et al., 2001; Dong et al., 2017; Gao et al., 2017; Zhang et al., 2018). Pancreas grafts from *hCD46* transgenic pigs survived 396 days after transplantation into non-human primates (van der Windt et al., 2009). Kidney grafts from GTKO*/hCD55* pigs survived for 499 days (Kim et al., 2019). Heart grafts from GTKO*/hTBM/hCD46* gene-modified pigs survived for 945 days (Mohiuddin et al., 2016). These results suggest that inhibition of the complement response is another effective way of inhibiting hyperacute rejection.

Other methods can also be used to inhibit hyperacute rejection. For example, the level of α-Gal can be reduced by transducing enzymes that compete with α-1, 3-galactosyltransferase (Sharma et al., 1996). In another study, specific siRNA was used to reduce the expression of α-1, 3 galactosyltransferase *via* RNA interference (Zhu et al., 2005). Plasmapheresis can decrease the level of α-1, 3 galactosyltransferase, which reduces the risk of activation of the complement response and delayed hypersensitivity (Watts et al., 2000).

### 2.2 Acute vascular rejection

Acute vascular rejection usually occurs two to three days after xenotransplantation. However, a detailed mechanism for this response remains unclear. This rejection may be caused by the interaction between the xenograft and the recipient’s xenoantibodies, macrophages, or platelets. The combination of XNAs and xenograft endothelial causes the activation of xenograft endothelial cells and receptor of macrophage, which induces the expression of various specific proteins, including cytokines, endothelial adhesion molecules, and blood coagulation factors. These factors can cause inflammation, thrombosis, cellulose precipitation or diffuse blood clotting in the xenograft, which lead to xenograft loss or inactivation (Gollackner et al., 2004). At present, the main strategies for addressing acute vascular rejection are to inhibit the activities of endothelial cells and macrophage, induce the expression of anticoagulants on the surface of porcine organs, and inhibit NF-kB signaling.

1) Inhibition of activity of endothelial cells and macrophage. Endothelial cells and macrophage release a variety of cytokines after activation. These cytokines participate in inflammation, thrombosis, and coagulation formation. Inhibition of macrophages and endothelial activities helps alleviate acute vascular rejection. Meanwhile, human leukocyte antigen G (HLA-G), a non-classical MHC Ⅰ molecule, is mainly expressed in the extravillous trophoblast cells of the maternal-fetal barrier and plays an significant role in maintaining maternal immune tolerance to the fetus as well as normal pregnancy. In xenotransplantation, the soluble HLA-G can protect porcine endothelial cells from NK cell; thus, *HLA-G* transgenic pigs are a potential option as donors in xenotransplantation (M. H. Zeng et al., 2006). The fibroblasts from the GTKO/*HLA-G5* pigs showed enhanced resistance to complement-mediated lysis ability (Zhou et al., 2022). HLA-E/human beta2-microglobulin transgenic pigs showed the protection against xenogeneic human anti-pig natural killer cell cytotoxicity (Weiss et al., 2009). Signal regulatory protein α (SIRPα), a major macrophage inhibitory receptor, can inhibit the macrophages activity when it binds to CD47. Porcine cells that expressing human CD47 could almost completely resist phagocytosis by macrophages (Ide et al., 2007).
2) Induction of the expression of anticoagulants on the surface of porcine organs. Coagulation dysfunction is an important cause of acute vascular rejection. The expression of one or more anticoagulant substances on the surface of pig organs by genetically modified methods, can significantly inhibit platelet aggregation and activation after xenotransplantation, thus alleviating the acute vascular rejection (Crikis et al., 2010). The same effect can be achieved by reducing the expression of procoagulant substances.
3) Inhibition of NF-kB signaling. Endothelial adhesion factors, cytokines and procoagulant factors mostly function through the NF-kB signaling pathway and participate in the formation of inflammation, thrombosis, and coagulation. The A20 protein can regulate the NF-kB expression, inhibit NF-kB activation and nuclear transfer, prevent endothelial cells activation and prevent the production of related inflammatory molecules such as vascular cell adhesion molecules and interleukins, thus inhibiting acute vascular rejection (Ferran, Stroka, Badrichani, Cooper, & Bach, 1997; Oropeza et al., 2009).

### 2.3 Acute cellular rejection

Hyperacute rejection and acute vascular rejection are mainly related to humoral immune system, whereas acute cellular rejection is primarily based on cell immunity. The most important cells in acute cellular rejection are NK cells and T lymphocytes. After xenotransplantation, antigen presenting cells present xenoantigen epitopes to recipient CD4^+^ T Cells *via* xenogeneic MHC class Ⅱ molecules. The xenoantigen of the graft can also be presented to CD8^+^ T cells *via* the MHC class I molecules of the recipient. The proliferation of CD4^+^ T cells and CD8^+^ T cells induces the production of interleukin-2 (IL-2) and interferon-γ (IFN-γ), which induces a series of immune rejection reactions (Maksoud, 2004). At present, T cell response is mainly inhibited by multigenic modifications that reduce acute cellular rejection. Pigs with multigenic GTKO/hCRPs modification were originally developed to overcome primary humoral immunity, however, Gal epitope loss and hCRP expression on porcine cell surfaces can also slow down the T cell proliferation and some cytokine mediated responses, thereby inhibiting acute cellular rejection to a certain extent (Xu, Goodman, Sasaki, Lowell, & Mohanakumar, 2002; Saethre et al., 2008). Cytotoxic effects mediated by human CD8^+^ T Cells, especially CD8^+^ CTL cells, mainly occur through the Fas/FasL apoptosis pathway. Kawamoto et al. overexpressed human FasL and Fas in pig islet cells, and found that the overexpression interfered with the apoptotic pathway, resulting in resistance to the cytotoxic effect mediated by CD8^+^CTL cells (Kawamoto et al., 2006). Recombinant hCTLA4-Ig expressed in piglets induced a 50% reduction in the proliferative response of human T lymphocytes, thus specifically and effectively inhibiting cellular and humoral immunity through negative immune regulation (Martin et al., 2005). Badin et al. transplanted the neuronal cells from *hCTLA4-Ig* transgenic pigs into Parkinson monkeys and observed long-term clinical recovery in the monkeys (Badin et al., 2010). Hara et al. found that the knockdown of CⅡTA, an MHC class Ⅱ transactivator, can effectively reduce the expression of MHC class Ⅱ molecules in pigs, thereby slowing down the stimulation of CD4^+^ T cells and reducing xenoantibody-mediated injury and T cell response (Hara et al., 2013).

### 2.4 Chronic rejection

To date, the chronic rejection in immune disorders has not been convincingly explained. Chronic rejection usually occurs months or years after xenotransplantation and mainly proceeds through humoral immunity, which is mediated by XNAs and the complement system. The low immune response of XNAs in the circulatory system leads to perivascular inflammation and injury to the vascular endothelium of the xenograft, which is accompanied by proliferation of vascular smooth muscle cells that block the blood vessels, and eventually leads to arteriosclerosis and xenograft loss. Chronic rejection may be inhibited by transducing new anti-inflammatory genes, such as *TFPI* (tissue factor inhibitor), *ENTPD1* (*CD39*), and *hHO-1* (human heme oxidase), to GTKO/hCRPs pigs. Of course, it still needs to be strengthened in overcoming chronic rejection before xenotransplantation comes into clinical applications.

Except for reduction or prevention the attack by the human immune system, how to improve the activity of transplanted organ is also a key concern. As reported, deletion of p53 or overexpression of BCL2 could inhibit cell apoptosis (Masaki et al., 2016; Olbrich et al., 2017; Zhang et al., 2020), which could provide a new different approach to inhibit exnograft cell apoptosis or prolong xenograft cell survival.

## 3 Interspecies chimeras

Another potential organ source is by creating human organs from human pluripotent stems cells (PSCs) in animal bodies *via* forming interspecies chimeras. Chimeras refer to cells in an organism that are derived from two different zygotes. This phenomenon usually occurs during embryonic development and does not involve immune rejection (or good immune tolerance). Chimerism has been reported in humans under natural conditions (such as in twins) (Dunsford et al., 1953) or during *in-vitro* fertilization (IVF) (Strain, Dean, Hamilton, & Bonthron, 1998). In scientific research, the intraspecies chimerism in lower animals, including drosophila, *xenopus*, zebrafish, birds and other model animals, was first applied in studies on development issues. As an important tool for studying the developmental biology, the use of intraspecies mice chimeras made a very significant contribution to the study of cell fate determination, cell migration, immune system development, organogenesis and stem cells. Therefore, intraspecies chimeras were originally used as a convenient biomarker to study embryonic development (Tarkowski, 1961; Le Douarin and Jotereau, 1975; Le Douarin, Jotereau, Houssaint, & Belo, 1976).

The first reported interspecies chimera that survived to adulthood was a chimera from a goat and a sheep (Fehilly, Willadsen, & Tucker, 1984). That study showed the feasibility of generating interspecies chimeras. In 2001, Mercer et al. reported that human-mouse chimeric liver animals were used as an animal model to study hepatitis C virus infection *in vivo*. Although a high ratio of chimerism was observed in the liver, chimerism in small animals can only be used for human cell level research, and is difficult to use in human organ transplantation (Mercer et al., 2001). In terms of large animals, in 2006, Zeng et al. developed a “human-goat chimera” by injecting bone marrow stem cells at the early stage of goat embryonic development (F. Zeng et al., 2006). However, this process was challenging to do in large animals and easily caused miscarriage, resulting a very low ratio of exogenous cells in animal organs obtained was very low. Only a particular population of cells (e.g., hematopoietic stem cells) can be studied, thus making targeting a specific organ difficult.

With the developments in stem cell research and regenerative medicine, an increasing number of researchers have attempted to use stem cells, particularly patient-derived iPS cells to generate human organs or tissues *in vitro*, which is also one of the ultimate goals of regenerative medicine. In 2016, mouse-rat hetero-diploid embryonic stems cells (ESCs) have been successfully constructed using mouse and rat haploid ESCs. These ESCs had a stable hetero-diploid genome and were able to differentiate into all three germ layers and early germ cells (Li et al., 2016). Given the high complexity of organogenesis, Kobayashi et al. adopted the blastocyst complementation method to mimic the certain microenvironments *in vivo* (Kobayashi et al., 2010). PSCs isolated from a normal mouse or rat were injected into the blastocysts of defective pancreatic development *Pdx1* ^*−/−*^ mice and retransplanted into the uterus of surrogate mice. The newborn mice had a chimeric pancreas and showed normal pancreas function. In 2012, Usui et al. used the same method to obtain allogeneic source compensation in blastocysts from defective renal development *Sall1* ^*−/−*^ mice (Usui et al., 2012). By injecting mouse PSCs into *Pdx1* ^*−/−*^ deficient rat blastocysts, Yamaguchi et al. generated rat-sized pancreata composed of mouse-PSC-derived cells. Islets from these mouse-rat chimaeric pancreata were transplanted into diabetes mice. The transplanted islets successfully normalized and maintained host blood glucose levels for over 370 days in the absence of immunosuppression (Yamaguchi et al., 2017). Aside from the pancreas, many other organs have been generated by blastocyst complementation, including kidney, thymus, blood and vascular endothelial cells, lungs and bronchi, and gametes (Isotani, Hatayama, Kaseda, Ikawa, & Okabe, 2011; Hamanaka et al., 2018; Goto et al., 2019; Mori et al., 2019; Kobayashi et al., 2021). Blastocyst complementation could also generated exogenic pancreas *in vivo* in apancreatic cloned pigs (Matsunari et al., 2013). These studies showed the potential of using xenogeneic animals to generate human organs for clinical application.

However, numerous scientific issues that remain to be addressed before organs can be generated from interspecies chimeras for clinical application: (1) Embryonic mortality is high, and interspecies chimeras such as the *Sall1* ^*−/−*^ mice rarely survive to adulthood. (2) The main barriers to interspecies chimeras, which may be related to the evolutionary distance between species, have not been fully elucidated. (3) Organs are multicellular structures that are organized in three dimensions. Thus, the mechanisms involved in the organ development are immensely complex and multifaceted. (4) The sizes and other morphological characteristics of the chimeric organs are determined by the recipient blastocyst animal, Thus limiting the selection of large animals for clinical application. (5) Primate stem cells are different from rodent stem cells, and their developmental potential, chimerism ability and chimerism timing remain to be determined (Nichols and Smith, 2009). The ratios of chimerism between human ES cells and chicken embryo or human ES cells and mouse blastocyst have been very low (Goldstein, Drukker, Reubinoff, & Benvenisty, 2002; James, Noggle, Swigut, & Brivanlou, 2006). Reports on chimera in rhesus monkeys suggest that these animals require a different approach from that on mice because their cells are heterogeneous at the early stage of blastocyst development (Tachibana et al., 2012). Exhilaratingly, Tan et al. first generated the human-monkey chimeric embryos in 2021, which developed strategies to improve human chimerism in evolutionarily distant species (Tan et al., 2021). Yu et al. derived PSCs from mice, horses, and humans that are permissive for direct PGC-like cell induction *in vitro* and are capable of contributing to intra- or inter-species chimeras *in vivo* (Yu et al., 2021). Currently, many species have been used for the research of interspecies chimerism, including non-human and human related researches (Table 2).

**TABLE 2:** Interspecies chimerism in different species.

	Chimeric species	Research objects	References
Non-human	Japanese quail-chick	Thymuses and bursa development	Le Douarin and Jotereau, (1975); Le Douarin, Jotereau, Houssaint, and Belo, (1976)
	Goat-sheep	Reproductive incompatibilities	Fehilly, Willadsen, and Tucker, (1984)
	Mouse-rat	Chimeric pancreas	Kobayashi et al. (2010)
	Mouse-rat	Thymus	Isotani, Hatayama, Kaseda, Ikawa, and Okabe, (2011)
	Mouse-rat	Chimeric renal development	Usui et al. (2012)
	Mouse-rat	Hetero-diploid ESCs	Li et al. (2016)
	Mouse-rat	Chimeric pancreata	Yamaguchi et al. (2017)
	Mouse-rat	Pluripotent stem cell-derived mouse kidneys	Goto et al. (2019)
	Monkey-pig	Functional cells derived from monkey	Fu et al. (2020)
	Mouse-rat	Gametes	Kobayashi et al. (2021)
Human related	Human-mouse	Hepatitis C virus infection	Mercer et al. (2001)
	Human-chicken	Integration and differentiation of human embryonic stem cells	Goldstein, Drukker, Reubinoff, and Benvenisty, (2002)
	Human-goat	Multitissue engraftment of human primitive hematopoietic cells and their differentiation	F. Zeng et al. (2006)
	Human-pig	Hepatocytes	Fisher et al. (2013)
	Human-pig	hPSCs derived progenies	Wu et al. (2017)
	Human-monkey	Chimeric competency of human extended pluripotent stem cells	Tan et al. (2021)

Pigs have always been a good choice as an animal source for xenotransplantation. Chimeric pigs are obtained by injecting inner cell masses into blastocysts (Nagashima, Giannakis, Ashman, & Nottle, 2004). Human-pig chimerism has been reported in human liver cells that had been grafted into pigs *via* intrauterine injection and postnatal injection; the proteins produced by human hepatocytes can be detected in pigs after 6 weeks (Fisher et al., 2013). Of course, such simple grafting, rather than regular chimerism, can produce only certain proteins made by human liver cells, not human cell-derived organs for xenotransplantation. Wu et al. found that instead of naïve hPSCs, an intermediate hPSC type exhibited higher capability to generate differentiated progenies in post-implantation pig embryos (Wu et al., 2017). Fu et al. demonstrated that domesticated cynomolgus monkey embryonic stem cells exhibited the capability to integrate and differentiate into functional cells in a porcine model (Fu et al., 2020). The use of pig-human chimerism still has a long way to go before it can be used in clinical xenotransplantation.

## 4 Endogenous virus infection in pigs

As an alternative to allotransplantation, xenotransplantation using pig cells, tissues, or organs has made tremendous progress in recent years. However, the clinical use of porcine organs has been hindered by the potential risk of transmission of pig viruses to humans. Although many known potential viruses and microorganisms can be removed by strict feeding conditions, porcine endogenous retroviruses (PERV) are unavoidable. PERV is incorporated into the pig genome in the form of proviral DNA and replicates as the cells proliferate. PERV was integrated into the pig genome more than six million years ago, and formed three subtypes (PERV-A, PERV-B and PERV-C). Two subtypes of PERV-A and -B can be found in the genome of all pigs, and both of which can infect human cells (Patience, Takeuchi, & Weiss, 1997; Takeuchi et al., 1998). By contrast, PERV-C does not ubiquitously appear in the pig genome and only infects pig cells (Jung et al., 2013). PERVs are harmless to pigs, however, whether they are potential harmful to humans remains unclear. PERVs have been verified to be transmitted from pig to human *in vitro* (Denner et al., 2013). Since xenotransplant recipients are under strong immunosuppression, it is desirable to avoid PERV infection.

Scientists have developed various strategies to reduce the risk of PERV transmission to human recipients. These strategies include inhibition of PERV expression using a PERV-specific vaccine (Denner, Mihica, Kaulitz, & Schmidt, 2012; Waechter and Denner, 2014), antiretroviral drugs (Demange et al., 2015; Argaw, Colon-Moran, & Wilson, 2016; Denner, 2017), PERV-specific small interfering RNAs (Ramsoondar et al., 2009; Semaan, Kaulitz, Petersen, Niemann, & Denner, 2012) and PERV gene knockout by genome editing technologies (Semaan, Ivanusic, & Denner, 2015; Yang et al., 2015; Niu et al., 2017). In 2015, Denner et al., 2003 reported that they failed to knock out of PERVs in pig PK15 cells using ZFN technology (Semaan et al., 2015). They found that ZFN is extremely toxic to the transfected cells. In addition, they presumed that the cytotoxic effects are due to the specific cutting of a high copy number of the PERV gene or the non-specific cleavage of off-target sites, which both produce numerous double-stranded DNA breaks in the pig genome. In the same year, Yang et al. reported that they completely eradicated 62 copies of PERV in porcine PK15 using CRISPR/Cas9 (Yang et al., 2015). Two years later, their team successfully generated the PERV knockout pigs (Niu et al., 2017). In 2021, Yang’s team generated an extensively edited pigs with inactivated endogenous retroviruses, thus enhancing the compatibility of pig organs with the human immune and coagulation system (Yue et al., 2021). In 2022, the multigene edited pig heart was given to a dying patient (David Bennett Sr). The new pig heart pumped for about 2 months after his transplant surgery, and eventually failed with the patient’s death. The cause of his death was not clear, however, the porcine cytomegalovirus (PCMV) were detected in his body, which may have contributed to his death. All these reports indicated that the potential risk of transmission of pig viruses to humans remains a big obstacle in the clinical use of porcine organs for xenotransplantation.

## 5 Prospects and challenges

Aside from the inevitable immune obstacles in xenotransplantation, numerous challenges also need to be addressed in the use of genetically modified pigs in clinical application. However, rapid developments in transgenic technology and recent remarkable progress in large-animal cloning, particularly the emergence of GTKO/hCRP genetically modified pigs, bring xenotransplantation a major step closer to clinical application. Although interspecies chimeras meet great challenges, we believe that future research developments will lead to the realization of animal-based transplantation medicine. At present, in some European and American countries, pig hearts, kidneys, and livers have reportedly been transplanted to patients that suffered from advanced organ failure. In 2020, GTKO pigs were approved by the United States Food and Drug Administration (FDA) to be a source of human therapeutics including xenotransplantation. In 2022, the university of Alabama reported that a brain-dead decedent was transplanted a kidney from 10-gene modified pig (Porrett et al., 2022). Furthermore, the University of Maryland Medical Center reported that an extensively edited pig heart was given to a dying patient. All these cases marked the clinical application of xenotransplantation from a pig to a human. Although the survival times of the xenografts were relatively short or xenotransplantation was used only as a transition to allotransplantation, hope remains for the use of xenogeneic organ transplantation in the future. The road toward the introduction of clinical xenotransplantation is proving long and arduous, but progress is steadily being made.
